# Disulfiram-induced cytotoxicity and endo-lysosomal sequestration of zinc in breast cancer cells

**DOI:** 10.1016/j.bcp.2014.12.014

**Published:** 2015-02-01

**Authors:** Helen L. Wiggins, Jennifer M. Wymant, Francesca Solfa, Stephen E. Hiscox, Kathryn M. Taylor, Andrew D. Westwell, Arwyn T. Jones

**Affiliations:** Cardiff School of Pharmacy and Pharmaceutical Sciences, Cardiff University, Redwood Building, Cardiff, Wales CF10 3NB, UK

**Keywords:** Breast cancer, Disulfiram, Lysosomes, Zinc, Fluozin-3

## Abstract

Disulfiram, a clinically used alcohol-deterrent has gained prominence as a potential anti-cancer agent due to its impact on copper-dependent processes. Few studies have investigated zinc effects on disulfiram action, despite it having high affinity for this metal. Here we studied the cytotoxic effects of disulfiram in breast cancer cells, and its relationship with both intra and extracellular zinc. MCF-7 and BT474 cancer cell lines gave a striking time-dependent biphasic cytotoxic response between 0.01 and 10 μM disulfiram. Co-incubation of disulfiram with low-level zinc removed this effect, suggesting that availability of extracellular zinc significantly influences disulfiram efficacy. Live-cell confocal microscopy using fluorescent endocytic probes and the zinc dye Fluozin-3 revealed that disulfiram selectively and rapidly increased zinc levels in endo-lysosomes. Disulfiram also caused spatial disorganization of late endosomes and lysosomes, suggesting they are novel targets for this drug. This relationship between disulfiram toxicity and ionophore activity was consolidated via synthesis of a new disulfiram analog and overall we demonstrate a novel mechanism of disulfiram-cytotoxicity with significant clinical implications for future use as a cancer therapeutic.

## Graphical abstract

**Figure:**
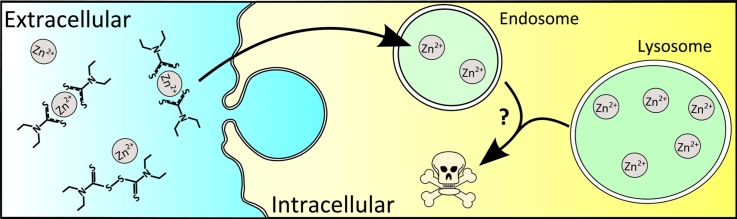

## 1 Introduction

Many current cancer therapies are limited by the severity and frequency of adverse side effects and there is high demand for non-toxic alternatives. One source of new therapies may be through repurposing of clinically approved drugs, where safety in patients has already been demonstrated. Disulfiram has a long medical history as an alcohol deterrent, however more recently has demonstrated anti-cancer effects in a range of solid and hematological malignancies [1]. The biological activity of disulfiram is attributed to its ability to bind divalent cations and consequently disrupt metal dependent processes, particularly those involving copper and zinc [2], [3]. Observations that both these metal ions are involved in oncogenic development have led to increased interest in the anti-cancer potential of this drug [4]. As part of a copper complex, disulfiram has been reported to induce apoptosis in both cultured breast cancer cells and xenografts through proteasomal inhibition [5], [6], [7]. These complexes have also been shown to stabilize the NFκB inhibitor protein, IκB, thus re-sensitizing gemcitabine resistant tumors with enhanced NFκB signaling [8]. In a case study of a patient with stage IV ocular melanoma with liver metastases, combination therapy involving disulfiram and zinc gluconate was able to induce remission with almost no side effects [9]. These observations have led to its introduction to clinical trials, including one involving patients with hepatic malignancies treated with disulfiram and copper gluconate (NCT00742911, University of Utah). Additionally, disulfiram treatment has been reported to remove essential copper and zinc ions from enzymes that regulate extracellular matrix degradation and oxygen metabolism resulting in suppression of cancer invasion and angiogenesis in vitro and in vivo [2], [3].

**Fig. 1:**
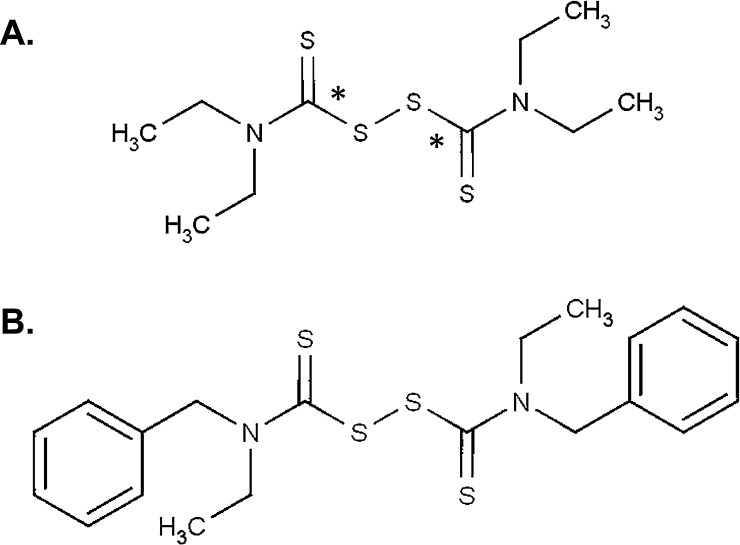
Structure of disulfiram and the disulfiram analog FS03EB. (A) *Indicates metal binding regions within the structure. (B) ^1^H NMR (500 MHz, CDCl_3_) d 1.30 (3H, bs, CH_3_), 1.47 (3H, s, CH_3_), 4.05 (4H, bs, CH_2_CH_3_), 5.26 (2H, s, CH_2_Ph), 5.41 (2H, s, CH_2_Ph), 7.39 (10H, m, ArH); ^13^C NMR (125 MHz, CDCl_3_) d 11.12 (CH_3_), 13.20 (CH_3_), 47.18 (CH_2_), 52.04 (CH_2_), 55.80 (CH_2_), 59.54 (CH_2_), 127.48 (ArCH), 127.72 (ArCH), 128.20 (ArCH), 128.49 (ArCH), 128.79 (ArCH), 128.99 (ArCH), 134.57 (ArC), 135.24 (ArC), 198.82 (C=S), 195.33 (C=S); MS (EI^+^) *m*/*z* 420.08 (M^+^); HR-MS (ESI^+^) *m*/*z* [M+H]^+^ calculated 421.0895, found 421.0896.

Much of the current literature surrounding disulfiram focuses on its capacity to bind copper ions, via two metal binding regions in its structure (Fig. 1A). Relatively little has been done to determine the role of zinc in its anti-cancer properties despite the fact that it also has high affinity for this metal [3]. Studies have highlighted the role of zinc in the etiology of breast cancer where high expression of zinc transporter proteins such as ZIP7 and ZIP10, in breast cancer cell models increases intracellular zinc levels and is associated with endocrine therapy resistance and increased invasiveness [10], [11]. Additionally, zinc has been reported to increase pro-survival signaling [12] and inhibit caspases [13] in vitro. Taken together these reports suggest that high zinc levels promote cancer cell survival. Paradoxically, high intracellular zinc is also associated with oxidative toxicity, implying that the cell maintains tight homeostatic control of this metal and that drugs which dysregulate this fine balance may induce toxicity [14]. As the concentration of zinc is higher in cancerous compared to non-cancerous breast tissue [15] it is possible that drugs which alter intracellular zinc levels would be selectively toxic to cancer cells.

In this study we investigate the role of both intra and extracellular zinc in the anti-cancer activity of disulfiram. We demonstrate the effect of zinc and copper on the cytotoxicity of the drug across a panel of cancerous and non-cancerous breast cell lines. We describe a novel mechanism of action for disulfiram, via its ability to rapidly increase intracellular zinc levels in endo-lysosomal compartments and alter the subcellular localization specifically of late lysosomal structures. Both these effects potentially impact on lysosome function. Interestingly, zinc levels in a non-cancerous breast cell line remain unaltered by disulfiram treatment and taken in the context of the literature surrounding zinc dysregulation in breast cancer, our results demonstrate a selective effect of disulfiram that may have significant clinical implications for its future clinical use.

## 2 Materials and methods

### 2.1 Chemicals and reagents

Disulfiram, diethyldithiocarbamate (DDC), sodium pyrithione, DMSO, Na-HEPES, NH_4_Cl, Triton X-100, BSA, ZnCl_2_, CuCl_2_, cholera toxin, insulin, epidermal growth factor, and hydrocortisone were obtained from Sigma–Aldrich (Dorset, UK). Disulfiram, DDC, and sodium pyrithione were dissolved in DMSO to produce a stock concentration of 10 mM and stored at −20 °C. CellTiter blue viability reagent was purchased from Promega (Southampton, UK). Anti-EEA1 (#6104490), anti-LAMP2 (#H4B4), and anti-LC3B (#2775) antibodies were obtained from BD Bioscience (Oxford, UK), Developmental Studies Hybridoma Bank (Iowa, USA) and Cell Signaling Technology (MA, USA) respectively. RPMI, FBS, DMEM/F12, horse serum, Fluozin-3, Hoechst 33342, dextran-Alexa 647 (10 kDa), Alexa-488 (A-11001) and Alexa-546 (A-11010) conjugated anti-mouse and anti-rabbit antibodies were from Life Technologies (Paisley, UK).

### 2.2 Synthesis of disulfiram analog

FS03EB (bis(N-benzylethylthiocarbamoyl)disulphide; Fig. 1B) was synthesized according to the method of Liang et al. [16]. Briefly, N-benzylethylamine and carbon disulphide (2:1 molar ratio) were mixed together in the presence of carbon tetrabromide (one equivalent) in dimethylformamide as solvent at room temperature. Following purification by column chromatography, the identity and purity of the product was confirmed using NMR spectroscopy and mass spectrometry [17]. FS03EB was then dissolved in DMSO to produce a stock concentration of 10 mM.

### 2.3 Cell culture

MDA-MB-231, MCF-7, T47D, and BT474 were maintained in RPMI 1640 supplemented with 10% FBS. MCF-10A cells were maintained in DMEM/F12 supplemented with 5% horse serum, 100 ng/ml cholera toxin, 10 μg/ml insulin, 20 ng/ml epidermal growth factor, and 500 ng/ml hydrocortisone [18]. Herein these are respectively termed complete media. All cell lines were obtained from ATCC and routinely tested for mycoplasma infection.

### 2.4 Viability assays

To account for different growth rates, cells were seeded in black 96-well plates at densities that provided 70% confluency after 72 h. After a minimum of 24 h, cells were treated with disulfiram, disulfiram metabolite DDC, FS03EB or DMSO ± copper or zinc supplements for the indicated time points. Viability studies were conducted using the CellTiter Blue assay according to manufacturer's protocol. All viability studies were conducted in complete media.

### 2.5 Live cell imaging of intracellular zinc

Microscopy analysis was conducted on a Leica SP5 confocal inverted microscope equipped with a 488 nm laser and 40× objective using Leica LAS AF software. For this, cells were preloaded with 5 μM Fluozin-3 diluted in cell imaging media (phenol red free RPMI media supplemented with 10% FBS and 50 mM Na-HEPES pH 7.4) for 30 min, before being washed thrice with PBS which was then replaced with 1 ml cell imaging media. In live cells representative region of interest was captured before and subsequent to addition of a 1 ml solution of 10 μM disulfiram, sodium pyrithione (positive control) or diluent control. Images are displayed as a multiple projection of 10 *z*-planes through the cells.

### 2.6 Flow cytometry

Cells were preloaded with Fluozin-3 for 30 min as above and treated with disulfiram, DDC, FS03EB, sodium pyrithione or diluent control in cell imaging media or serum free imaging media (phenol red free RPMI supplemented with 50 mM Na-HEPES pH 7.4) ±20 μM zinc or copper for 10 min. Following trypsinization, cells were resuspended in PBS, and centrifuged three times at 150 × *g*. Cells were then resuspended in media, and 10,000 events were analyzed via flow cytometry using a BD Biosciences FACSVerse system equipped with a 488 nm laser.

### 2.7 Comparative localization of intracellular zinc with endocytic probes in disulfiram treated cells

To label the entire fluid-phase endocytic network, MCF-7 cells were incubated for 4 h with 2.5 mg/ml dextran-Alexa 647 diluted in cell imaging media. To specifically label lysosomes, cells were incubated with dextran-Alexa 647 for 2 h followed by a 4 h chase [19]. During the final stages of this incubation, cells were incubated with Fluozin-3 for 30 min, washed with PBS, and treated with 10 μM disulfiram for 10 min. Cells were then washed three times with PBS, and analyzed via live cell confocal microscopy.

### 2.8 Localization of endocytic organelles and induction of autophagy in disulfiram treated cells

MCF-7 cells were treated with 1 μM disulfiram or equivalent diluent control for 3 h (for endosomes and lysosomes) or 24 h (for autophagosomes) before being washed in PBS, fixed and permeabilised by either −20 °C methanol for 10 min (for LAMP2) and LC3B labeling) or with 3% PFA for 15 min, 50 mM NH_4_Cl for 10 min and 0.2% Triton X-100 for 5 min (for EEA1 labeling). After fixation the cells were washed three times in PBS, incubated for 1 h in blocking buffer (2% FBS, 2% BSA in PBS) then incubated for 1 h with primary antibody diluted 1:200 (LAMP2 and EEA1) or 1:400 (LC3B) in blocking buffer. The cells were then washed three times in PBS before being incubated for 1 h with secondary antibodies and Hoechst 33342 (1 μg/ml). Following a further three washes with PBS they were mounted in oil. Imaging was conducted via confocal microscopy for LAMP2 and EEA1. For LC3B imaging was conducted on a Leica DMIRB inverted epi-fluorescent microscope, equipped with a 40× objective.

### 2.9 Statistical testing

For all studies three independent experiments were conducted in triplicate (for viability studies) or duplicate (for flow cytometry studies) and significance of data determined, as appropriate, using students two tailed *T*-test in Microsoft Excel and displayed as *p < 0.05 or **0.001. Data is presented as the mean and standard error of the mean. Co-localization via microscopy was determined using JaCOP plugin of ImageJ and the Pearsons coefficient was used as a measure of the ratio of pixels which were labeled with dextran-Alexa 647 and Fluozin-3 where 1.0 is complete co-localization [20]. Pearson's coefficient is expressed as ±standard error of the mean.

## 3 Results

### 3.1 Disulfiram produces a biphasic cytotoxic response in some breast cancer cell lines

**Fig. 2:**
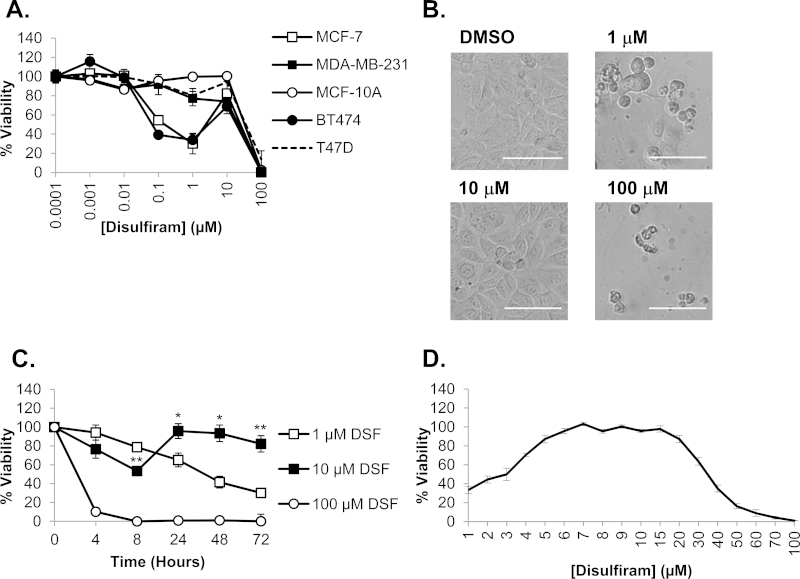
The cytotoxic profile of disulfiram in breast cancer cell models. (A) Cells were treated with a serial dilution of disulfiram in complete media and viability analyzed after 72 h. (B) MCF-7 cells were imaged using brightfield microscopy following 72 h disulfiram (DSF) treatment. Scale bar shows 100 μm. (C) MCF-7 cells were treated for 8–72 h with disulfiram prior to analyzing viability. *T*-tests were conducted between equivalent time points to compare 1 and 10 μM data, *p < 0.05, **p < 0.001. (D) MCF-7 cells were treated for 72 h with disulfiram at concentrations between 1 and 100 μM prior to analyzing viability. Error bars show standard error.

Initial cell viability experiments were conducted to investigate the sensitivity of a panel of breast cancer cell lines to disulfiram. These were chosen to model clinically relevant disease sub types, including estrogen receptor positive (ER^+^), human epidermal growth factor receptor 2 (HER2) negative (MCF-7 and T47D), ER^+^/HER2^+^ (BT474) and ER^−^/HER2^−^ (MDA-MB-231) and the non-cancerous breast epithelial MCF-10A line. Disulfiram over 72 h was only toxic to ER^+^ cells (MCF-7 and BT474, IC_50_ 0.3 μM vs. MDA-MB-231 IC_50_ and MCF-10A IC_50_ > 10 μM; Fig. 2A), however not all ER^+^ cells responded equally to the drug (T47D IC_50_ > 10 μM) demonstrating that the presence of ER is not a prerequisite for sensitivity. In disulfiram responsive cells (MCF-7 and BT474) cytotoxicity was biphasic, producing a recovery peak at 10 μM with almost complete restoration of viability. The biphasic effect in MCF-7 cells was confirmed by microscopy showing clear morphological damage at 1 μM that is consistent with loss of cell viability. These effects were absent at 10 μM where morphology was comparable to diluent controls; increasing the disulfiram concentration to 100 μM then restored the toxic 1 μM phenotype (Fig. 2B).

To further investigate this biphasic response we determined whether it was affected by disulfiram concentration at different incubation times. MCF-7 cells were treated with 1, 10 and 100 μM disulfiram over a range of time points and cell viability was then determined. Despite an initial cytotoxic phase at <8 h, cell viability at 10 μM was restored at greater than 24 h (Fig. 2C). For other concentrations disulfiram produced a time dependent decrease in viability; at 1 μM viability steadily decreased between 4 and 72 h whereas at 100 μM there was a rapid loss of viability to <10% within 4 h. This data demonstrates that the 10 μM disulfiram response is due to recovery from initial effects that are not manifest as cell death but rather a reduction in metabolic rate as determined by this assay. When the biphasic peak in MCF-7 cells was investigated at concentrations between 1 and 100 μM at a single 72 h time point, viability was restored to >80% between 5 and 20 μM concentrations of the drug (Fig. 2D).

### 3.2 Disulfiram specifically increases intracellular zinc levels in breast cancer cells

**Fig. 3:**
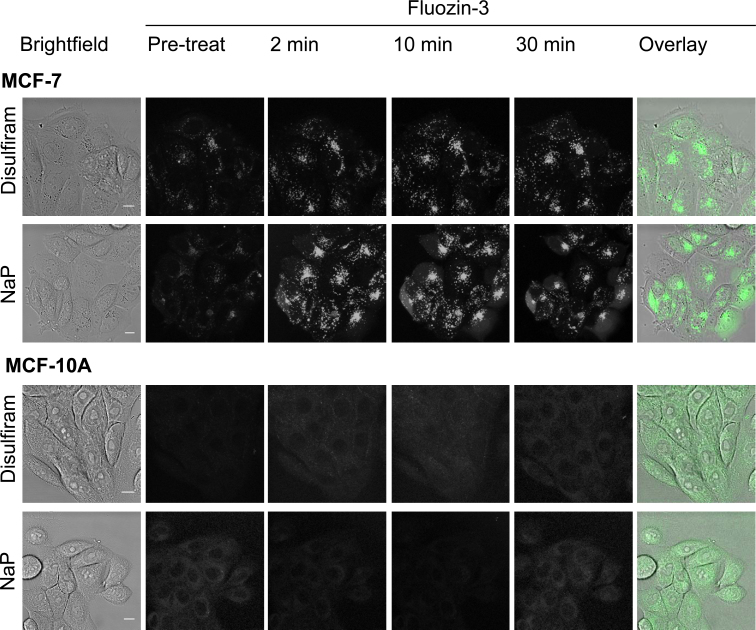
Disulfiram selectively increases intracellular zinc levels in punctate structures of breast cancer cells. Cells were preloaded with Fluozin-3 for 30 min and imaged before (pre-treat) and subsequent to the addition of 10 μM disulfiram or sodium pyrithione (NaP) in cell imaging media. Images are multiple *z*-projections from a series of 10 equally spaced, single projections and are representative from three independent experiments. Scale bars show 10 μm.

To investigate the relationship between disulfiram and intracellular zinc levels we employed live cell confocal microscopy using the zinc probe Fluozin-3. In complete media, disulfiram rapidly (<10 min) increased intracellular zinc levels in MCF-7 cells, specifically to label punctate compartments, to levels comparable to those obtained using the well established zinc ionophore sodium pyrithione (Fig. 3). A significant proportion of sodium pyrithione treated cells were also noted for displaying diffuse cytosolic Fluozin-3 labeling whereas zinc in disulfiram treated cells was only observed in punctate structures. Surprisingly, zinc levels remained unaffected by either disulfiram or sodium pyrithione treatment in the non-cancerous MCF-10A cell line.

**Fig. 4:**
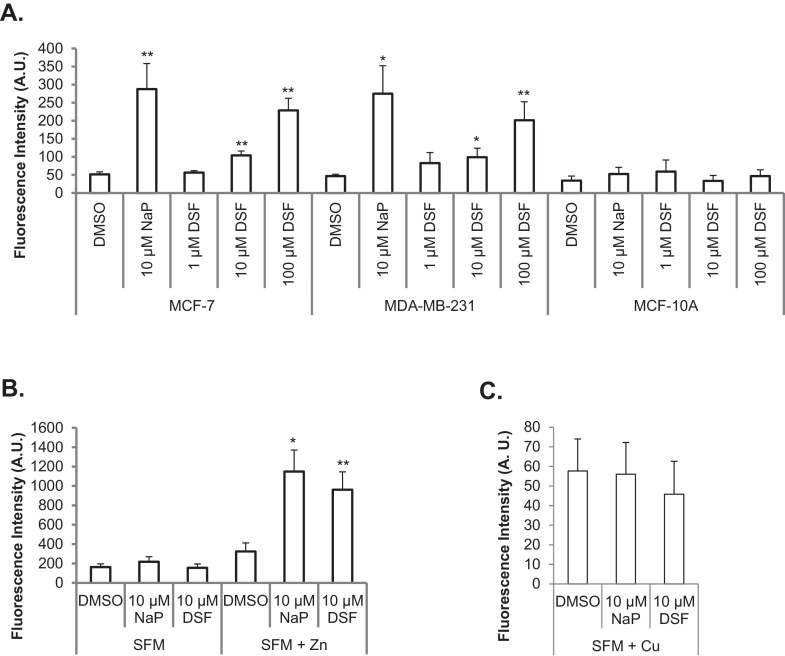
Disulfiram delivers extracellular zinc into cells rather than releasing intracellular stores. Cells were preloaded with Fluozin-3 and treated with disulfiram (DSF), DMSO or sodium pyrithione for 10 min in complete media (A) or serum free media (SFM) ±20 μM zinc (B) or copper (C) in MCF-7 cells. Fluozin-3 fluorescence was then determined via flow cytometry. Error bars show standard error. T-tests in (A) were conducted between DMSO and treatment groups and in (B, C) were conducted between SFM and SFM + Zn/Cu for each treatment, *p < 0.05, **p < 0.001.

In order to further investigate the disulfiram effects seen by microscopy, a flow cytometry assay was developed to enable quantitative comparison of intracellular zinc levels in disulfiram and sodium pyrithione treated cells. The data supported the microscopy findings as 10–100 μM disulfiram significantly increased intracellular zinc levels in both MCF-7 and MDA-MB-231 cell lines (Fig. 4A), while zinc levels in MCF-10A cells remained unaffected by the same treatment. To further investigate this, and minimize the effects of extracellular zinc in serum, the flow cytometry experiments were conducted in serum free media- low zinc and copper conditions. Under these conditions, neither sodium pyrithione nor disulfiram evoked a statistically significant increase in intracellular zinc in MCF-7 cells (Fig. 4B). Supplementation of serum free media with 20 μM zinc was sufficient to completely restore, and in fact exaggerate, the ionophore ability of both disulfiram and sodium pyrithione, demonstrating that this ionophore activity is dependent on extracellular zinc levels. This effect, with respect to the selectivity of the dye for zinc versus copper which could possibly also provide Fluozin-3 fluorescence, was tested by conducting the experiments in serum free media supplemented with copper. Here copper was unable to significantly restore the fluorescence of Fluozin-3 in disulfiram or sodium pyrithione treated cells (Fig. 4C), demonstrating that the increased fluorescence of Fluozin-3 observed in Fig. 3, Fig. 4 was specifically due to the effects of zinc.

### 3.3 Disulfiram sequesters intracellular zinc in endo-lysosomal compartments

**Fig. 5:**
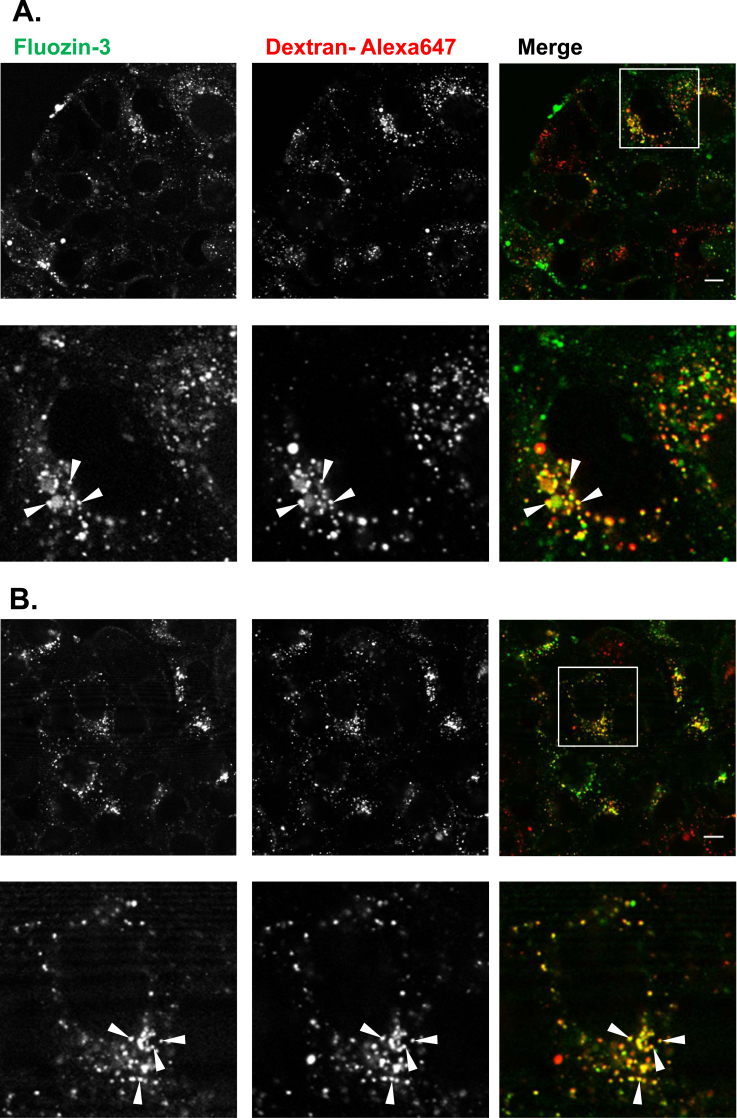
Disulfiram increases intracellular zinc in endo-lysosomal compartments of breast cancer cells. Dextran-Alexa 647 was used to highlight late endo-lysosomal compartments (A) or the entire fluid phase endocytic network (B) in MCF-7 cells, as described in Section 2. Cells were then incubated with Fluozin-3, treated with 10 μM disulfiram for 10 min and co-localization between Fluozin-3 and dextran-Alexa 647 was analyzed via confocal microscopy. Images show single *z*-projections through the cells and lower panel in (A) and (B) show a zoomed image of an identified cell in upper panel. Images shown are representative from three independent experiments. Co-localization is marked by arrow heads. Scale bars show 10 μm.

The observation that disulfiram sequestered zinc in punctate structures lead us to investigate the nature of these compartments. In order to determine whether they were components of the endocytic network, fluorescent dextran was utilized as an endocytic probe for co-localization studies. By conducting a 2 h pulse with dextran-Alexa 647 followed by cell washing and a further 4 h chase, the probe can be trafficked and confined to lysosomes [19]. Dextran pulse-chase experiments were performed and cells were co-stained with Fluozin-3 and treated with disulfiram; the degree of co-localization between dextran-Alexa 647 and Fluozin-3 was then analyzed using live cell confocal microscopy. Fig. 5A demonstrates that a significant portion of dextran labeled lysosomes were also labeled with Fluozin-3 and very few dextran only structures were observed (Pearsons coefficient = 0.49 ± 0.06 for three independent experiments). When the entire fluid phase network was labeled with dextran as a single 4 h pulse (Fig. 5B), a higher degree of co-localization was observed between the two probes (Pearsons coefficient = 0.67 ± 0.04) suggesting that disulfiram was also driving zinc into earlier compartments of the endocytic network.

**Fig. 6:**
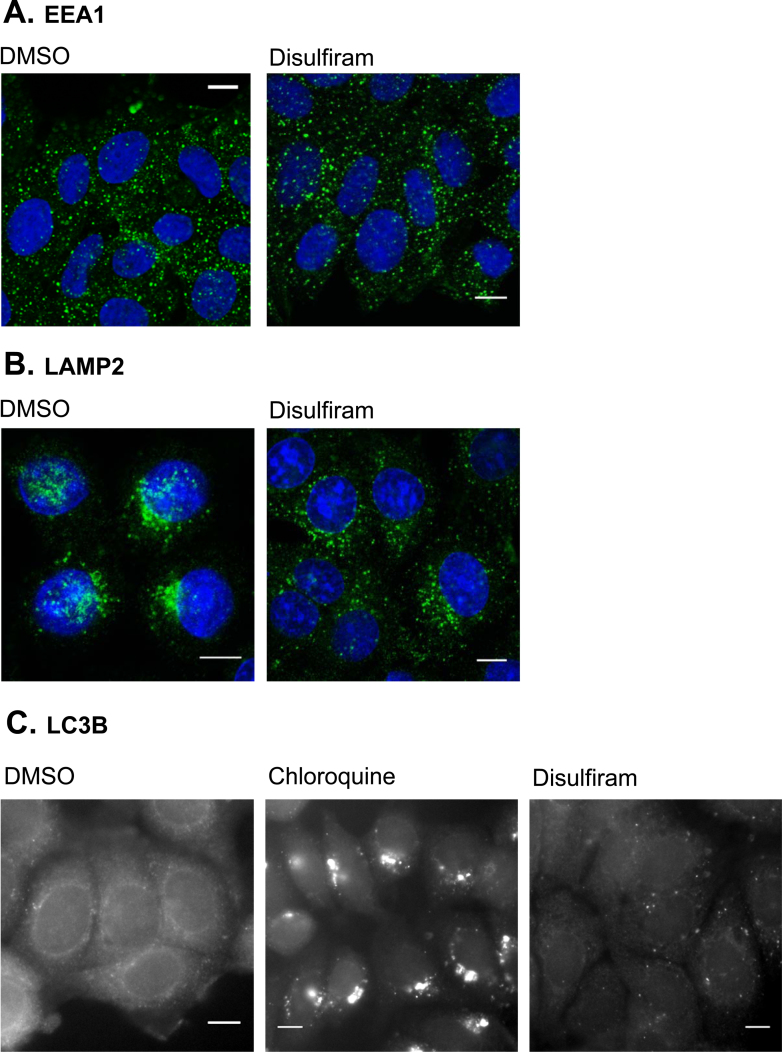
Disulfiram causes mislocalization of lysosomes, however does not alter localization of early endosomes or autophagic membranes. MCF-7 cells were treated with 1 μM disulfiram or chloroquine (100 μM, 6 h) and then analyzed via immunofluorescence microscopy using antibodies recognizing EEA1 (A), LAMP2 (B) or LC3B (C). Blue nuclei are labeled with Hoechst 33342. Image shows single projection through the middle of the cell and is representative from three independent experiments. Scale bars show 10 μm.

To investigate whether disulfiram influences the spatial organization of these endocytic structures MCF-7 cells were treated with the drug for 3 h prior to performing immunofluorescence analysis using antibodies recognizing the early endosomal marker, Early Endosome Antigen 1 (EEA1) or the late endosomal/lysosomal marker, Lysosome Associated Membrane Protein 2 (LAMP2) [21]. Disulfiram was observed to have no effect on the localization of early endocytic structures (Fig. 6A), however caused late endosomes and lysosomes to be redistributed from typical perinuclear clusters observed in control cells to more diffuse scattering throughout the cytoplasm (Fig. 6B). The ability of disulfiram to induce autophagy was investigated using antibodies recognizing a marker of autophagic membranes, microtubule-associated Light Chain 3 B (LC3B). For this experiment cells were either treated with disulfiram for 24 h prior to LC3B immunofluorescence analysis, or for 6 h with chloroquine, an agent which causes accumulated LC3B via stabilization of autophagosomal membranes [22]. Chloroquine treated cells displayed large LC3B containing structures representing autophagosomes, however this phenotype was absent in cells treated with disulfiram (Fig. 6C).

### 3.4 Supplementation with zinc or copper increases disulfiram potency

**Fig. 7:**
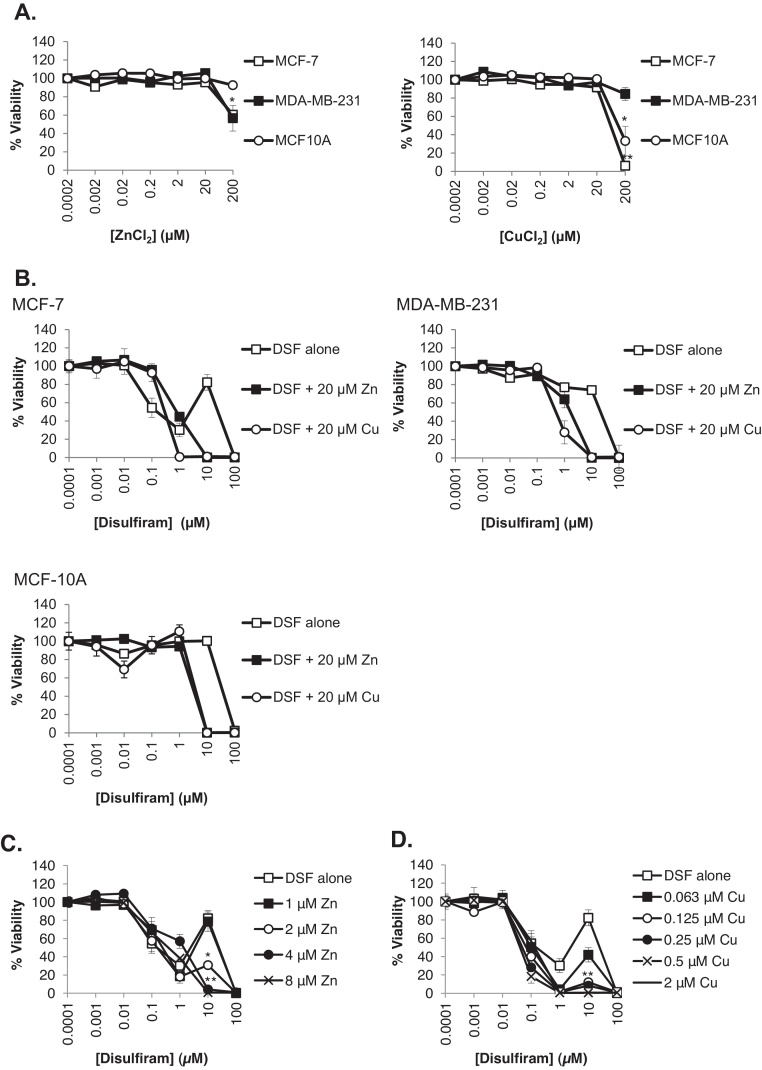
Zinc and copper enhance the cytotoxicity of disulfiram. (A) Cells were treated with a serial dilution of zinc or copper for 72 h in the presence of complete growth media before cell viability analysis was performed. (B) Cells were treated with disulfiram ±20 μM zinc or copper in complete media for 72 h prior to performing viability analysis. (C and D) Viability of MCF-7 cells treated with disulfiram ± zinc or copper in supplemented in complete media was analyzed after 72 h. Error bars show standard error. *p*-values for data in Fig. 7B are provided in Table 1.

The interaction between disulfiram and zinc observed in our previous results lead us to consider whether enrichment of complete media with zinc or copper could affect the cytotoxicity of the drug in cancerous and non-cancerous breast cells. As control experiments, we initially investigated whether supplementing cell media with increasing concentrations of zinc or copper in the absence of disulfiram had any effect on cell viability. These studies demonstrated that ≤20 μM zinc or copper was without effect but toxicity was observed at higher concentrations of both metals with MCF-7 and MCF-10A showing particular sensitivity to copper (Fig. 7A). When a non-toxic dose of zinc or copper (20 μM) was given in combination with disulfiram, cytotoxicity was significantly enhanced in all cell lines (Fig. 7B, Table 1). In the case of MCF-7 cells, the disulfiram biphasic response was completely abolished by both zinc and copper supplementation, however cytotoxicity of disulfiram at lower concentrations was reduced by addition of either metal supplement. The minimum concentration of copper and zinc supplement required to influence cell recovery (biphasic peak) in MCF-7 cells was then determined and data in Fig. 7C and D shows that 2.0 μM zinc and 0.125 μM copper significantly reduced the ability of the cells to recover from disulfiram effects. At higher concentrations both metals completely reversed the biphasic response.

**Table 1:** Co-incubation of copper/zinc significantly altered the cytotoxico profile of disulfiram.

	MCF-7				MDA-MB-231				MCF-10A			
[DSF]	0.1 μM	1 μM	10 μM	100 μM	0.1 μM	1 μM	10 μM	100 μM	0.1 μM	1 μM	10 μM	100 μM
DSF alone	54.5 ± 10.5	30.2 ± 7.5	82.2 ± 8.7	0.1 ± 0.2	91.6 ± 2.8	77.0 ± 10.8	74.0 ± 12.3	0.02 ± 0.1	95.5 ± 1.6	99.8 ± 1.6	100.3 ± 2.9	2.4 ± 1.2
DSF + Zn	95.8 ± 4.2**	44.6 ± 3.5*	0.1 ± 0.2**	0.1 ± 0.3	89.1 ± 4.6	63.9 ± 9.3	0.1 ± 0.1**	0.02 ± 0.1	93.3 ± 3.9	94.3 ± 2.9	0.3 ± 0.1**	0.3 ± 0.1
DSF + Cu	92.8 ± 9.8*	0.6 ± 0.2**	1.0 ± 0.4**	0.7 ± 0.2*	98.5 ± 2.2	27.9 ± 12.5*	0.7 ± 0.1**	1.0 ± 0.2*	95.7 ± 9.5	110.7 ± 7.1	0.16 ± 0.1**	0.3 ± 0.1

Breast cells were treated with the indicated concentrations of disulfiram ±20 μM zinc or copper for 72 h prior to performing viability analysis. Values displayed are percentage viability and standard error of the mean. *T*-tests were conducted between disulfiram treatment alone and disulfiram + zinc/copper.

* *p* < 0.05

** *p* < 0.001.

### 3.5 Cytotoxicity of disulfiram, diethyldithiocarbamate (DDC) and a structural analog correlates with ionophore activity

**Fig. 8:**
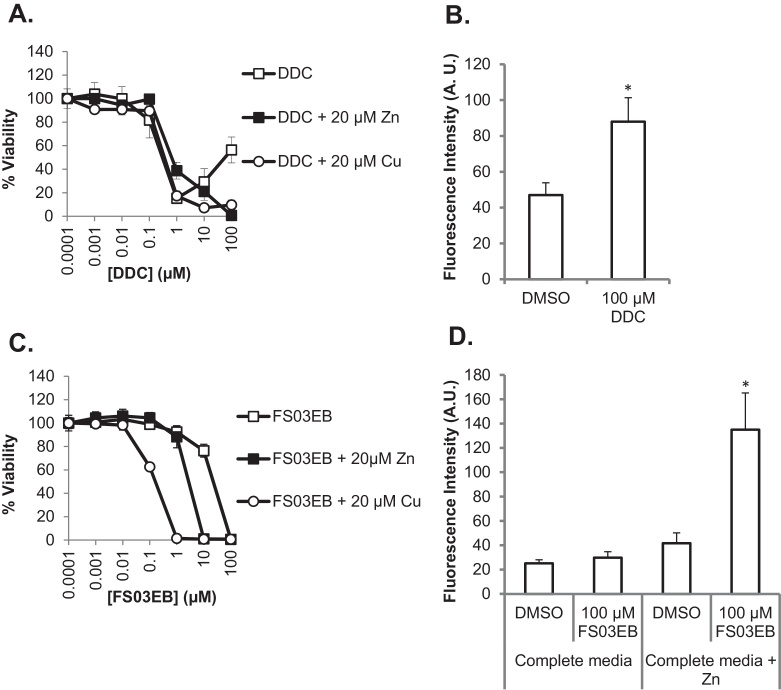
Toxicity of DDC and FS03EB correlates with zinc ionophore activity. (A, C) MCF-7 cells were treated with DDC (A) or FS03EB (C) ±20 μM copper or zinc in complete media for 72 h prior to performing viability analysis. (B, D) MCF-7 cells were preloaded with Fluozin-3, treated with DDC (B) or FS03EB ±20 μM zinc (D) for 10 min prior to measuring Fluozin-3 fluorescence via flow cytometry. Error bars show standard error. *p < 0.05, *p*-values for data in (A and C) are provided in Table 2.

Disulfiram in vitro and in vivo is rapidly metabolized to give two molecules of DDC [23]. The possibility that this major metabolite was able to induce toxicity and also act as a zinc ionophore was also investigated in MCF-7 cells. DDC displayed a sharp increase in toxicity at concentrations higher than 0.1 μM with evidence of recovery being observed at 100 μM, rather than at 10 μM as in the case of disulfiram (compare Fig. 8, Fig. 2). Supplementation with copper and zinc completely ablated the DDC biphasic effects (Fig. 8A) and significantly enhanced its toxicity (Table 2). The metabolite was also able to increase intracellular zinc levels (Fig. 8B) but was a less potent zinc ionophore compared with the parent drug (Fig. 4A).

**Table 2:** Co-incubation of copper/zinc significantly altered the cytotoxic profile of DDC and FS03EB.

	[DDC]				[FS03EB]			
	0.1 μM	1 μM	10 μM	100 μM	0.1 μM	1 μM	10 μM	100 μM
No supplement	81.7 ± 15.1	15.3 ± 2.1	29.3 ± 11.1	56.4 ± 11.0	98.9 ± 3.1	92.4 ± 5.4	76.4 ± 1.1	0.7 ± 0.4
Zn supplement	99.5 ± 2.4**	38.8 ± 7.1**	21.1 ± 7.6	0.7 ± 0.1**	104.4 ± 4.6	88.2 ± 9.3	1.1 ± 0.2**	0.7 ± 0.2
Cu supplement	89.5 ± 4.4	17.3 ± 4.3	7.2 ± 2.8	9.7 ± 4.1*	63.4 ± 1.9*	1.5 ± 0.2**	0.8 ± 0.2**	0.8 ± 0.1

MCF-7 cells were treated with DDC or FS03EB ±20 μM zinc or copper for 72 h prior to performing viability analysis. Values displayed are percentage viability and standard error of the mean. Student two-tailed *T*-tests were conducted between DDC or FS03EB treatment alone and FS03EB or DDC + zinc/copper.

* *p* < 0.05.

** *p* < 0.001.

To determine how cytotoxicity may relate to the capacity of disulfiram and DDC to deliver zinc, a close structural analog of disulfiram, FS03EB, was synthesized and viability assays were performed with this compound. FS03EB lacked any significant toxicity at concentrations below 100 μM in complete media and a biphasic response was not observed (Fig. 8C). Here, the core zinc binding thiuram disulfide pharmacophore was retained, but two of the terminal ethyl groups were replaced by the related benzyl group (see Fig. 1B for chemical structure and physical characterization of FS03EB). In complete media, and contrary to the effects observed with disulfiram and DDC, data in Fig. 8D show that FS03EB was unable to significantly increase intracellular zinc levels. However, co-incubation of this compound (>1 μM) with 20 μM zinc or copper was toxic leading to complete loss of viability at 1 μM and 10 μM for copper and zinc respectively (Table 2). When the zinc ionophore activity of FS03EB was investigated in conditions which induced toxicity (complete media + 20 μM zinc), it resulted in a >3-fold increase in Fluozin-3 fluorescence.

## 4 Discussion

Much of the literature surrounding the anti-cancer properties of disulfiram focuses on its interaction with copper, particularly as a disulfiram-copper complex [6], [24]. In contrast, the effects of zinc on the drugs toxicity are under-reported, despite knowledge that this metal is dysregulated in breast cancer cells [15]. The aim of this study was to determine the role of intra- and extracellular zinc in the anti-breast cancer properties of disulfiram. We demonstrated that under normal growth conditions the drug is able to selectively kill MCF-7 and BT474 breast cancer cell lines, whilst having no effect at physiologically relevant concentrations on T47D, MDA-MB-231 and the non-cancerous breast epithelial MCF-10A cell line. In disulfiram sensitive cells a biphasic cell viability profile was produced, manifest at concentrations between 5 and 20 μM. The biphasic response has, to varying extents, previously been implied in other studies involving breast [25] and other cancerous cell lines [1], however the underlying cause and its clinical significance remains to be determined. To investigate this further we examined the time dependent toxicity of disulfiram at concentrations within this biphasic range, and demonstrated that the response is due to recovery of initially affected cells. This effect is highly time dependent and may explain why this is frequently reported in the literature at time points greater than 24 h [1], [25], [26], however to our knowledge, no studies have investigated the sensitivity of cells to disulfiram at shorter (<8 h) time points.

Studies have shown that supplementing media with zinc to increase intracellular levels, presumably through zinc channels, induces oxidative toxicity [14] and inhibits NFκB signaling [27]. Our findings demonstrate that under normal conditions (complete media) disulfiram selectively increases intracellular zinc in breast cancer cells and this may have numerous cellular effects with some leading to toxicity. However, the fact that disulfiram resistant cells (MDA-MB-231) are sensitive to the zinc loading activity of disulfiram suggests a complex link between viability and intracellular zinc. It is also apparent that ionophore-independent mechanisms could contribute to disulfiram cytotoxicity, as 1 μM disulfiram did not show a measurable increase in intracellular zinc levels in MCF-7 cells, however produced >50% decrease in cell viability. Previous studies have demonstrated that disulfiram releases zinc from proteins [28], raising the possibility that the source of this metal which accumulates inside drug treated cells may be from intracellular proteins. However, our studies show that the ability of the drug to increase intracellular zinc is dependent on the availability of extracellular levels of this metal, supporting the hypothesis that the drug is acting as a zinc ionophore. This finding could have far reaching clinical consequences as comparative studies between cancerous and non-cancerous breast tissue from the same patient have shown that the latter has elevated zinc levels [15]. Tumors may therefore provide a more favorable environment for disulfiram to induce zinc-associated toxicity by providing an increased source of zinc for the drug to exert its ionophore action.

The observation that disulfiram is able to increase endo-lysosomal zinc levels is previously unreported, and may have important implications in its selective anti-breast cancer effect. The possibility exists that the cell utilizes certain compartments as an intracellular pool of zinc [29], and we hypothesize that breast cancer cells treated with disulfiram experience a sudden increase in zinc which the cell compartmentalizes to endo-lysosomes in an attempt to buffer the excess. The cytoprotective distribution of excess zinc to lysosomes has recently been reported, however high lysosomal zinc sequestration was also able to induce apoptosis when lysosomal release mechanisms were compromised [30]. Additionally, increased lysosomal storage of zinc has been observed in cancer cells treated with clioquinol, another zinc ionophore [31]. High zinc levels led to lysosomal dysfunction, causing the release of lysosomal enzymes to the cytoplasm and consequently apoptosis. Whether this represents a mechanism underlying disulfiram action here is not yet known but currently under investigation. It has been established that intracellular localization of endo-lysosomal components is integral to their cellular function, for example starvation and altered intracellular pH have been shown to redistribute lysosomes between perinuclear and peripheral regions of the cell [32], [33]. Here, we show that disulfiram is able to alter the sub-cellular localization of endo-lysosomal components and by this mechanism may alter their function, providing more evidence that the lysosomal sequestration of zinc may promote lysosomal disruption. Other studies have noted that increased lysosomal zinc is required for inducing autophagy in tamoxifen treated MCF-7 cells [34]. Despite increasing lysosomal zinc levels, we found that disulfiram did not induce autophagy.

It has previously been reported that the addition of copper [5], [9], [24], [25], zinc [9] and other metal ions such as cadmium [35] to the extracellular media increases the potency of disulfiram across a range of cancer cell types. The incubation of copper to remove the biphasic phase has previously been reported [25] but here we demonstrate that zinc supplement has the same effect. Critically the ability of either metal ion to remove the biphasic peak implies that the toxicity of disulfiram at 10 μM is limited by the availability of copper and zinc in the media. When considering the ability of disulfiram to increase intracellular zinc and copper levels, the possibility exists that an increase in extracellular copper and zinc allows disulfiram to transport more of these metal ions into the cell, which accounts for the increased toxicity at this concentration. The low concentrations of either copper and zinc required to increase disulfiram potency, suggest that increasing the availability of either metal ion could be achievable in vivo with oral supplements to enhance the cytotoxic effects of the drug. However, data with the MCF-10A cell line suggests that such supplementation may adversely alter the selectivity of the drug for breast cancer cells and could cause non-specific toxicity.

In vivo disulfiram is rapidly and extensively metabolized in a systemic manner; the first degradation product is DDC and this metabolite is thought to be a major contributor in the clinical effects of the drug [36]. DDC has been shown to have toxicity against breast cancer cells in vitro and increases intracellular copper in other model systems [7], [37]. Additionally, a clinical trial has shown that adjuvant DDC is able to increase survival in patients at high risk of metastatic breast cancer [38]. We show here that DDC, albeit to a lesser extent than disulfiram was also able to increase intracellular zinc levels and this may be a mechanism behind its increased cytotoxicity in the presence of high extracellular levels of these metal ions.

Measurable cytotoxicity <100 μM, and zinc ionophore activity of the new disulfiram analog, FS03EB, was only observed with zinc supplementation. This provides a direct link between zinc ionophore activity and cytotoxicity, and supports our hypothesis that the observed toxicity profiles for disulfiram, DDC and FS03EB relate to their capacity to increase intracellular zinc. Overall we propose that extracellular zinc levels and ionophore activity should be given higher prominence when discussing the effects of disulfiram on cancer cells.
